# Tuning the electronic properties of boron nitride nanotube by mechanical uni-axial deformation: a DFT study

**DOI:** 10.1186/1556-276X-6-160

**Published:** 2011-02-21

**Authors:** Shin-Pon Ju, Yao-Chun Wang, Ting-Wei Lien

**Affiliations:** 1 Department of Mechanical and Electro-Mechanical Engineering, Center for Nanoscience and Nanotechnology, National Sun Yat-sen University, Kaohsiung, 804, Taiwan

## Abstract

The effect of uni-axial strain on the electronic properties of (8,0) zigzag and (5,5) armchair boron nitride nanotubes (BNNT) is addressed by density functional theory calculation. The stress-strain profiles indicate that these two BNNTS of differing types display very similar mechanical properties, but there are variations in HOMO-LUMO gaps at different strains, indicating that the electronic properties of BNNTs not only depend on uni-axial strain, but on BNNT type. The variations in nanotube geometries, partial density of states of B and N atoms, B and N charges are also discussed for (8,0) and (5,5) BNNTs at different strains.

## Introduction

In nanoscale materials, especially for nanotubes, numerous special properties depend on their ultra-small sizes. Carbon nanotubes (CNTs), discovered by Iijima in 1991 [1], have been a very promising one-dimensional material in nanoscience. Theoretical calculations and experimental measurements on carbon nanotubes have shown many exceptional properties that make CNTs promising for several proposed applications, such as high Young's modulus and electronic properties [1-6]. Boron nitride nanotubes (BNNTs) were theoretically predicted in 1994 and were synthesized experimentally in the following year [7]. BNNTs are a structural analogy to CNTs that instead alternate boron and nitride atoms to replace the carbon atoms in the hexagonal structure. Although CNTs and BNNTs have similar structures, their properties are quite different. For example, electronic properties of CNT are distinctly different from those of BNNTs because of the large ionicity of B-N bonds [2]. Another difference is that BNNTs have a much better resistance to oxidation in high temperature systems than CNTs [8]. Moreover, the BNNT is independent of the chirality and diameter and is a semiconductor with a wide band gap [9].

As BNNTs have many special mechanical, thermal, electrical, and chemical properties and have a large number of potential applications, such as in composite materials, hydrogen storage, and force sensors [10-13], many scientists have studied the properties of BNNTs and related material [2,14-18]. The hydrogen storage attracted much attention in recent years especially. Ma et al. [16] found that the structure of BNNTs is better able to store hydrogen at high temperature than CNTs, such that BNNTs can store 1.8 to 2.6 wt% at 10 MPa. In theoretical studies, Cheng et al. obtained that capability of hydrogen storage in single-walled boron nitride nanotube arrays (SWBNNTA) can be increased with the increase of distance between BNNTs. Zhao and Ding [11] indicated that several gas molecules (H_2_, O_2_, and H_2_O) dissociate and chemisorb on BNNT edges, and the adsorption of these molecules induces a charge transfer. Yuan and Liew [18] reported that boron nitride impurities will cause a decrease in Young's moduli of SWCNTs. Moreover, the effect of these impurities in zigzag SWCNTs is more significant because of the linking characteristics of an increase in electrons. Mpourmpakis and Froudakis [19] discovered that BNNTs are preferable to CNTs for hydrogen storage because of the ionic character of BNNTs bonds which can increase the binding energy of hydrogen. In addition, some methods have been shown to improve the efficiency of storage. An increase in the diameter of BNNT can increase the efficiency of hydrogen storage [20]. Further, Tang et al. [21] improved the concentration of hydrogen storage to 4.6 wt% by bending the BNNTs. BNNTs also have many great physical and chemical properties. Zhi et al. [14] found that MWBNNTs have the ability to form covalent bonds with ammonia and can act as a solute in an organic solution. Chen et al. [15] obtained the result that field-emission current density of an Au-decorated boron nitride nanotube (Au-BNNT) is significant enhanced in contrast to pure BNNTs. Chen et al. [22] used ball milling-annealing to synthesize BNNTs and found that the average resistivity of that is 7.1 ± 0.9 × 10^12 ^Ω. Chopra and Zettl [2] observed that the BNNT has the highest elastic modulus of 700-900 GPa in one-dimensional fibers.

Recent studies have shown that applying strain to a one-dimensional material will affect its electrical property. Shiri et al. discovered that the band gap of silicon nanowire (SiNW) can be affected under uni-axial tensile strain. They also found that the strain induced direct-to-indirect transition in the band gap of SiNW with different diameters [23]. Tombler et al. used theoretical and experimental approaches to study the effect of single-walled carbon nanotubes (SWNTs) with deformation on its electrical conductance. They found the electrical conductance of SWNT is obviously reduced as compared to SWCNT without deformation [24]. For the theoretical studies, Li et al. [25] demonstrated that the transport property of CNT with double vacancy is reduced under external force. The stress-strain curve of armchair CNTs shows a step-by-step increasing behavior, and the C-C bond length varies significantly at specific strain during the tensile process. Those changes are more apparent for the smaller-sized armchair CNT. Wang reported a structural transformation from zigzag (Z-type) to an unusual type of fourfold-coordinated (H-type) and to armchair (A-type) structure in the ultrathin SiCNTs under uni-axial compression [26]. Wu et al. [27] found that the radial deformation of BNNT significantly affects the H_2 _adsorption energy on BNNT. They presented the relationship between the H_2 _adsorption energy at different adsorption sites and the extent of radial deformation of BNNT.

In experimental part, Kaniber et al. [28] utilized the piezoelectric device to apply different uni-axial strains to CNT. They mounted the CNT on two Au pads (source and drain) of a piezoelectric stack. When different voltages were applied to the piezoelectric device, the axial length of CNT can be adjusted. For CNT with different uni-axial strains, they found that the electronic properties of CNT can be affected by the uni-axial mechanical deformation. From this experiment and references [23-28] it is obvious that besides the size and shape of nanomaterials, the electronic properties can be further adjusted by applying the mechanical deformation. Since BNNTs have some material properties superior to CNT, it is worth understanding how to adjust the electronic properties of BNNT by the mechanical deformation for further applications, such as hydrogen storage for fuel cell. Therefore, this study utilizes DFT to investigate armchair (5,5) and zigzag (8,0) single-wall BNNTs under different uni-axial loadings. The HOMO-LUMO gap, radial bucking variety, and bond length are adopted to discuss the relationship between the mechanical deformation and electronic properties for the two different chiralities.

### Simulation model

**Figure 1:**
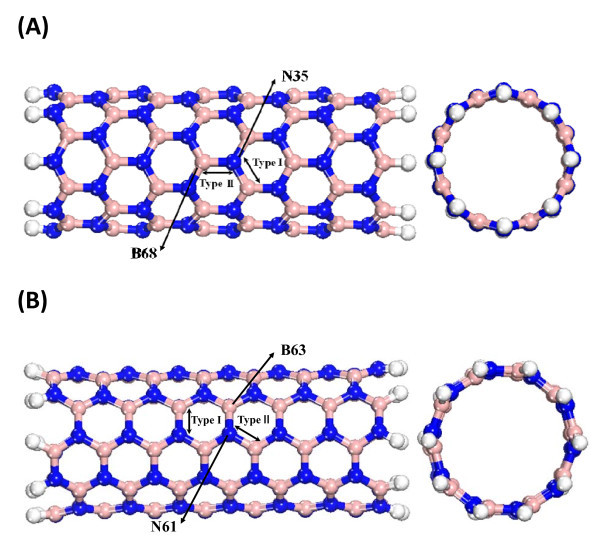
**Cross-section and side views of (a) single wall (8,0) BNNT and (b) (5,5) BNNT**. Gray, white, and blue beads stand for boron, nitrogen, and hydrogen atoms, respectively.

In this study, DFT methods are adopted to study the relationship between strain and electronic properties of single-wall armchair and zigzag BNNT. This method has been widely used in theoretical calculations of nanotube systems, including structural and electronic properties. Density functional semi-core pseudo-potentials (DSPP) [29] calculations were employed with double numerical basis sets plus d-functions (DND) and generalized gradient approximation (GGA) [30] with the Perdew-Wang 1991 (PW91) generalized gradient approximation correction [31]. Mulliken population analysis was used to obtain both the charge and net spin population on each atom. We chose the finite cluster (8,0) BNNT with length of 18.11 Å including totally 64 boron, 64 nitrogen, and 16 hydrogen atoms, and (5,5) BNNT with length of 18.25 Å including totally 70 boron, 70 nitrogen, and 20 hydrogen atoms as the studied systems. Table 1 lists the simulation result and compares it to the previous studies, Ref. [20]. The different profiles of bond type in (8,0) and (5,5) BNNT are shown in Figure 1a,b. The simulation result is close to other studies and means that our results are accurate.

**Table 1:** Diameter, bond length, and binding energy for different BNNTs

Nanotube and stoichiometry	Tube diameter	Bond length distribution (Å)		**Binding energy (eV/each atom**)
		Type I	Type II	
BNNT (4,4)	5.657	1.461	1.456	6.361
	5.49^a^	1.440^b^	1.444^b^	
BNNT (5,5)	7.043	1.457	1.455	6.422
	6.87^a^	1.439^b^	1.442^b^	
BNNT (6,6)	8.43	1.462	1.458	6.457
	8.23^a^	1.439^b^	1.440^b^	
BNNT (7,7)	9.49	1.462	1.459	6.476
	9.59^a^	1.438^b^	1.439^b^	
BNNT (8,8)	11.21	1.459	1.457	6.490
	10.95^a^	1.439^b^	1.439^b^	
BNNT (4,0)	3.556	1.499	1.439	6.187
	3.35^a^	1.476^b^	1.423^b^	
BNNT (5,0)	4.191	1.473	1.442	6.371
	4.08^a^	1.460^b^	1.429^b^	
BNNT (8,0)	6.263	1.460	1.454	6.606
	6.37^a^	1.445^b^	1.434^b^	

^a^From Ref. [20]; ^b^from Ref. [40].

## Results and discussion

**Figure 2:**
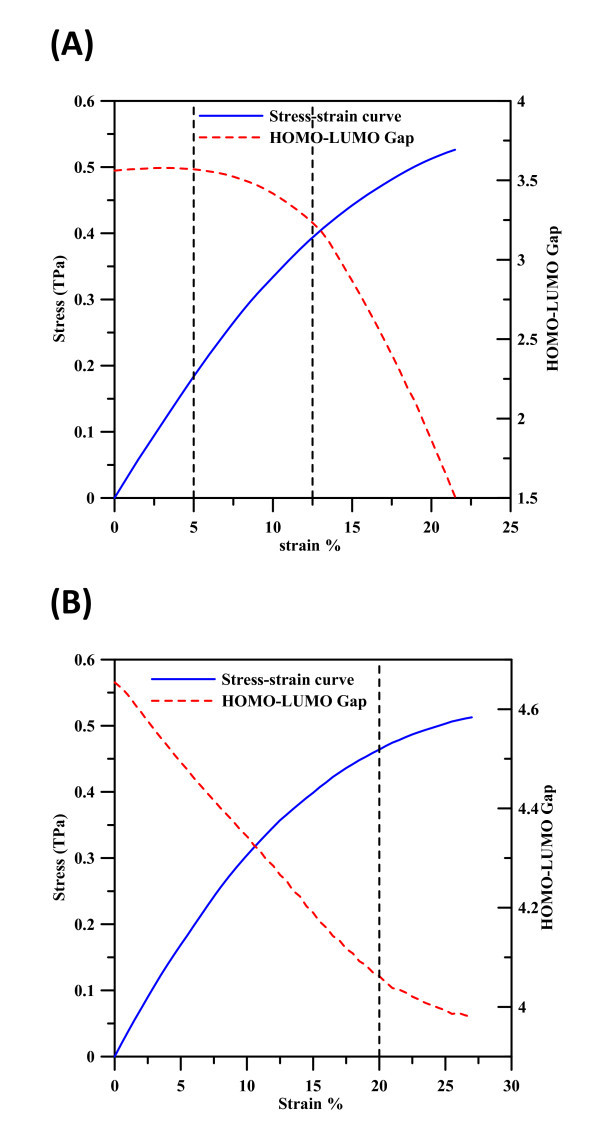
**Stress-strain profiles for (a) (8,0) Zigzag BNNT and (b) (5,5) Armchair BNNT**. The red line shows HOMO-LUMO gap variation at different strains.

In order to investigate material properties for armchair and zigzag BNNTs at different strains, (8,0) and (5,5) BNNTs of close radii are used. Although the results of other armchair and zigzag BNNTs are not shown in this study, the results are very similar for BNNTs of the same type. Figure 2 shows the profiles of axial stress and HOMO-LUMO (highest occupied molecular orbital and lowest unoccupied molecular orbital) gap at different strains for (8,0) armchair and (5,5) BNNTs. The stress on the *m *plane of the nanotube in the *n*-direction is calculated by [32].

(1) $$\sigma_{mn}=\frac{1}{N_{s}}\sum_{i}\left[\frac{mv_{i}^{m}v_{i}^{n}}{v_{i}}-\frac{1}{2v_{i}}{\vec{r}}_{i}\cdot {\vec{F}}_{i}^{\text{Int}}\right]$$

where *m *is the mass of atom *i*; $v_{i}^{m}$ and $v_{i}^{n}$ are the velocity components of atom *i *in the *m*- and *n*-directions, respectively; *v*_*i *_is the volume assigned around atom *i*; *N*_*s *_is the number of particles contained within region *S*, where *S *is defined as the region of atomic interaction; *r *is the position of atom *i*; and $F_{i}^{\text{Int}}$ is the internal force acting on atom *i*.

The first term on the right-hand side of Equation 1 describes the kinetic effect of the atomic motion and is dependent on the temperature. This term is not considered for our current DFT calculation. The second term expresses the effect of the interactive forces and is determined by the distance between the atoms. In Equation 2, *V*_*i *_is the Voronoi volume of atom *i *and is constructed by the perpendicular planes bisecting the lines between this atom and all of its neighboring atoms. Clearly, it is time-consuming to compute the Voronoi volume of each atom in the simulation system. Accordingly, Srolovitz *et al. *proposed the following formulation to obtain a sphere whose volume is equal to the original Voronoi volume [33]:

(2) $$\begin{array}{cc} V_{i}=\frac{4\pi }{3}a_{i}^{3} & a_{i}=\frac{\sum_{j}r_{ij}^{-1}}{2\sum_{j}r_{ij}^{-2}} \end{array}$$

where *a*_*i *_is the average radius of atom *i *and *r*_*ij *_is the distance between atom *i *and its neighboring atom *j*.

The normal strain in the axial direction of the BNNT is given by

(3) $$\varepsilon =\frac{\bar{l_{z(t)}}-l_{z(o)}}{l_{z(o)}}$$

where $\bar{l_{z(t)}}$ is the length of the BNNT in the axial direction following elongation and *l*_*z*__(__*o*__) _is the initial length, which the axial stress is zero after a complete geometry optimization by DFT. The stress-strain relationship of the BNNT can then be obtained from Equations 1 and 3.

The lengths of both (8,0) and (5,5) BNNTs after the relaxation by the DFT method are defined as the referenced lengths at strain of 0, where the axial stresses are 0 after calculation by Equation 1. As we focus on the electronic properties of the intact BNNTs at different strains without bond breakage, the maximal strains shown in Figure 2 before significant necking and some bond breakage are 21.5 and 27% for (8,0) and (5,5) BNNTs, respectively; the corresponding maximal stresses are about 0.526 and 0.511 TPa. For the stress-strain profiles, it is apparent that the stresses increase with an increase in strain in both cases. The profiles of HOMU-LUMO gaps, where the gap value for the (8,0) BNNT remains at a constant of 3.7 eV, are close to the reference value [34] from strain 0 to 5%, and then displays a parabolic decrease when the strain increases from 5 to 12.5%. As the strain is larger than 12.5%, the gap decreases linearly with the increase of strain. For (5,5) BNNT, the HOMO-LUMO gap is 4.65 eV at strain 0, which is close to the reference value [35], the gap linearly decreases with the increase of strain until the strain reaches 20%. When the strain is larger than 20%, the profile displays a parabolic decrease. Although the stress-strain profiles of (8,0) and (5,5) BNNTs seem very similar, the variations of HOMO-LUMO gaps at different strains are clearly different. Accordingly, Figure 2 clearly demonstrates that the mechanical deformations of BNNTs significantly influence their electronic properties, with the electronic properties of different chirality BNNTs displaying different responses to the strains. Further, different levels of strain may produce either linear or non-linear electronic property profiles.

**Figure 3:**
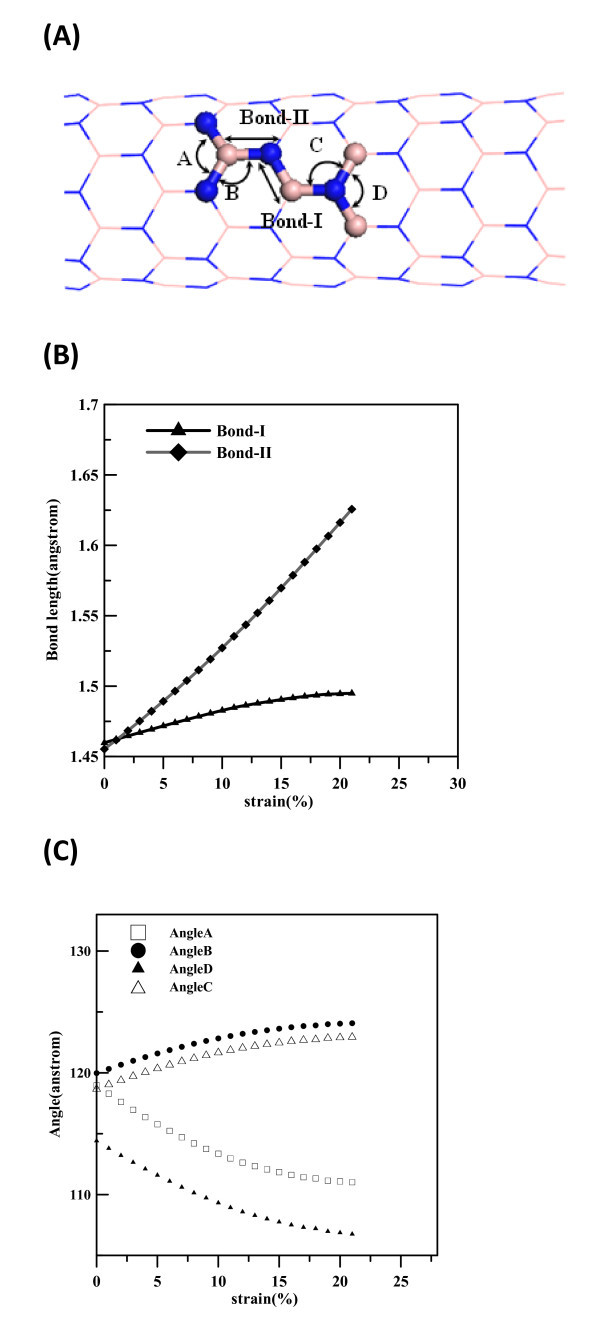
**Simulation model and definitions for (a) bond angles and bond lengths of (8,0) BNNT are shown**. Bonds parallel to axial are shown as Bond-II, and other ones slanted to the axial are shown as Bond-I. Bond angles are labeled as A, B, C, and D. **Variation of (b) **the radial buckling and bond lengths of (8,0) BNNT at different strains and **(c) **the radial buckling and bond angles of (8,0) BNNT at different strains.

The variations of bond lengths and bending angles of (8,0) BNNT at different strains are shown in Figure 3b,c, with the corresponding bond lengths and bending angles depicted in Figure 3a. The B-N bonds parallel to the axial direction are designated as Bond-II, and the B-N bonds slanted from the axial direction are labeled as Bond-I. According to the bending angles formed by different bond types and the central atom type, four angles labeled as A, B, C, and D are used to indicate different bending angle types used in Figure 3c. In Figure 3b, the lengths of Bond-I slightly increase with the increase of strain, but the lengths of Bond-II display a significant increase with the increase of strain. As shown in Figure 3c, angles B and C increase when the strain increases, whereas decreases in angles A and D can be seen as the strain increases. Consequently, the elongation of (8,0) BNNT is mainly due to the altering of bond angles and the elongation of Bond-II, which is parallel to the axial direction.

**Figure 4:**
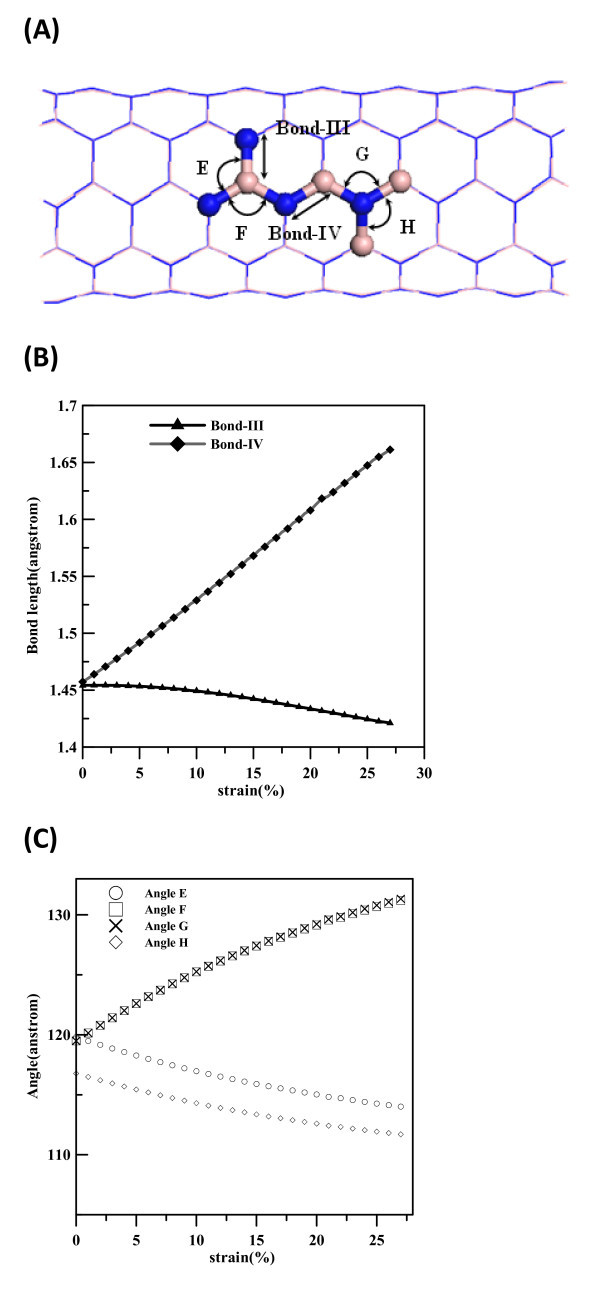
**Simulation model and definitions for (a) bond angles and bond lengths of (5,5) BNNT are shown**. Bonds normal to axial are shown as Bond-III, and other ones slanted to the axial are shown as Bond-IV. Bond angles are labeled as E, F, G, and H. **Variation of (b) **the radial buckling and bond lengths of (5,5) BNNT at different strains and **(c) **the radial buckling and bond angles of (5,5) BNNT at different strains.

The relationship of bond lengths and bending angles of (5,5) BNNT at different strains are shown in Figure 4b,c, with the corresponding bond lengths and bending angles depicted in Figure 4a. The B-N bonds normal to the axial direction are designated as Bond-III, and those slanted from the axial direction are labeled as Bond-IV. According to the bending angles formed by different bond types and central atom type, four angles labeled as E, F, G, and H are used to indicate different bending angle types in Figure 4c. In Figure 4b, the lengths of Bond-IV significantly increase with the increase of strain, but the lengths of Bond-III remain constant when the strain is smaller than 5% and slightly decrease when the strain is larger than 5%. As shown in Figure 4c, angles F and G increase when the strain increases, whereas decreases in angles E and H with the increase of strain can also be seen. Consequently, the elongation of the (5,5) armchair BNNT is mainly due to the altering of bond angles and the elongation of Bond-IV which is slanted from the axial direction.

**Figure 5:**
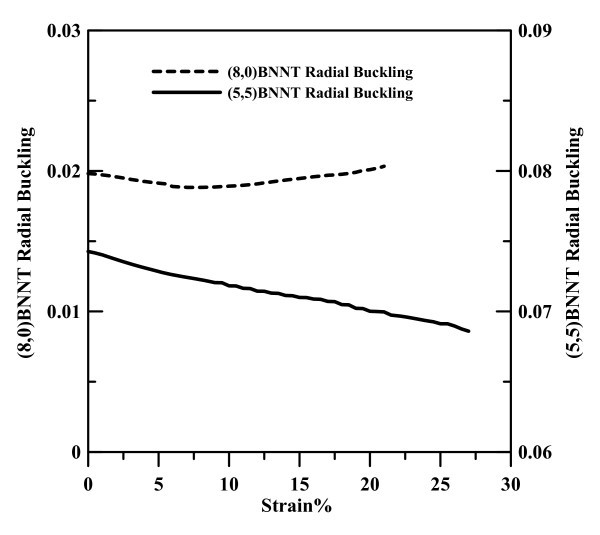
**Radial Buckling of (8,0) and (5,5) BNNT at different strains**.

In Figures 3c and 4c, at strain of 0 the bending angles D and H are about 113.9° and 116.5° for (8,0) and (5,5) BNNTs, respectively. The other three angles A, B, and C of (8,0) BNNT are close to 118.5° and angles E, F, and G of (5,5) BNNT are about 120°. N atoms and their nearest three B atoms form local pyramid structures and are not located on the same cylindrical surface, with N and B atoms occupying the outer and inner shells, respectively, as reported in previous studies [36]. This phenomenon is called radial buckling and can also be seen for SiC nanotubes and ZnO nanotubes [20,37]. To investigate the variation of radial bucking at different strains for (8,0) and (5,5) BNNTs, Figure 5 shows the radial buckling at different strains. The definition of radial buckling β is as shown in Equation 4:

(4) $$\beta =r_{N}-r_{B}$$

where *r*_B _and *r*_N _represent the radii of the B and N cylinders. If the value of radial buckling approaches zero, the B and N atoms will be located on the cylindrical surface of the BNNT, while a positive value indicates that the BNNT consists of two cylindrical surfaces with N atoms situated on the outer surface [36]. At strain of 0, the values of radial buckling are about 0.02 and 0.074 for (8,0) and (5,5) BNNTs, indicating the radial buckling is less significant for a zigzag BNNT. In Figure 5, the radial buckling of the (5,5) BNNT dramatically decreases with an increase in strain, indicating that the B and N atoms are gradually forced to the same cylindrical surface when the (5,5) armchair BNNT is subjected to an increasing uni-axial external stress. However, for the (8,0) zigzag BNNT, the value of radial buckling remains at an almost constant 0.02 when the strain continuously increases.

**Figure 6:**
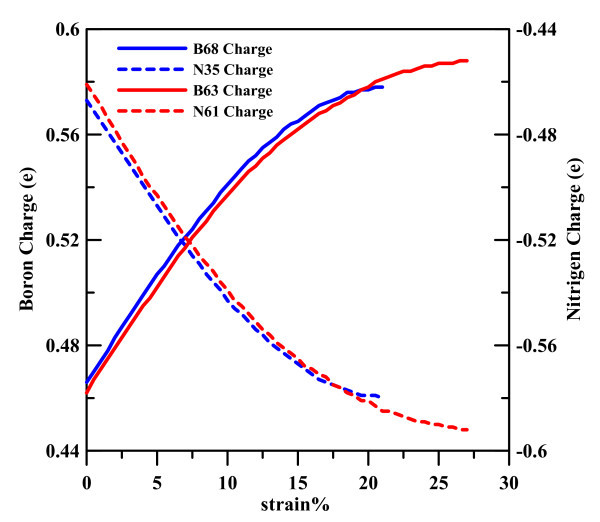
**Variation of the calculated atom charge of (8,0) and (5,5) BNNT. The solid and dashed lines show charge variation of boron and that of nitrogen, respectively**.

Figure 6 shows the Mulliken charges at different strains for the B63 and N61 atoms of the (8,0) BNNT and for the B68 and N35 atoms of the (5,5) BNNT. These B and N atoms are located in the central sections of the BNNTs, as shown in Figure 1; it is clear that the charge variations of B and N atoms at different strains are very similar for (8,0) and (5,5). At strain 0, the charges of B and N atoms are about 0.465 and -0465 eV, respectively, which are in agreement with previous studies [38]. When the strain becomes larger, the B and N atoms appear more ionic.

**Figure 7:**
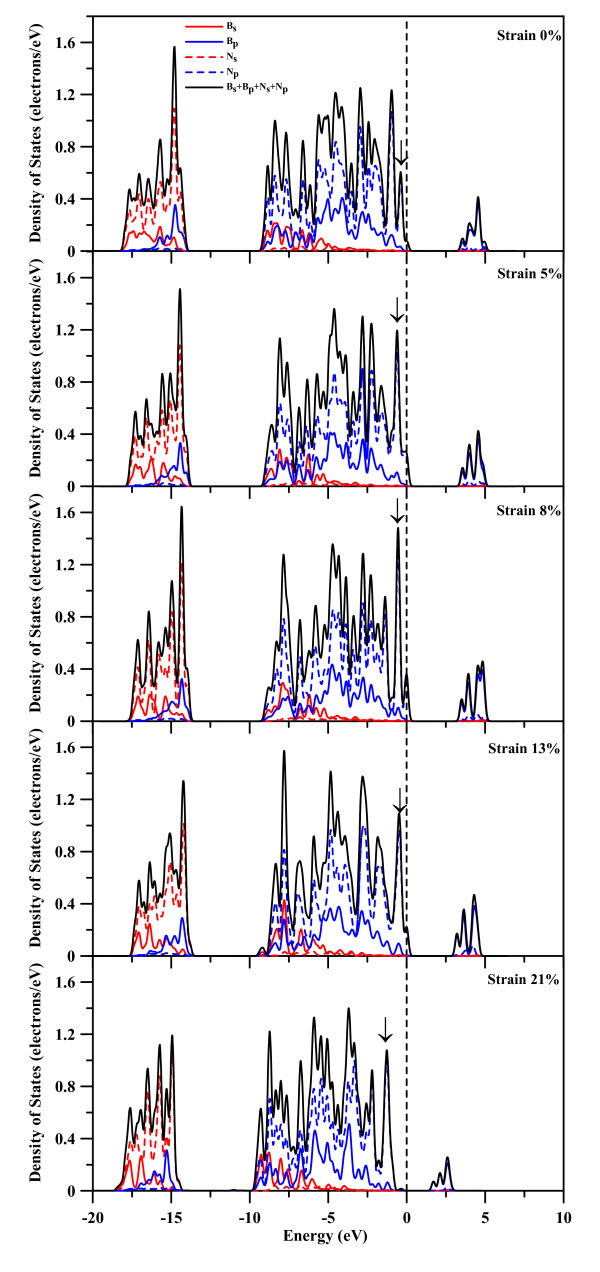
**PDOS profiles of B68 and N35 atoms**.

**Figure 8:**
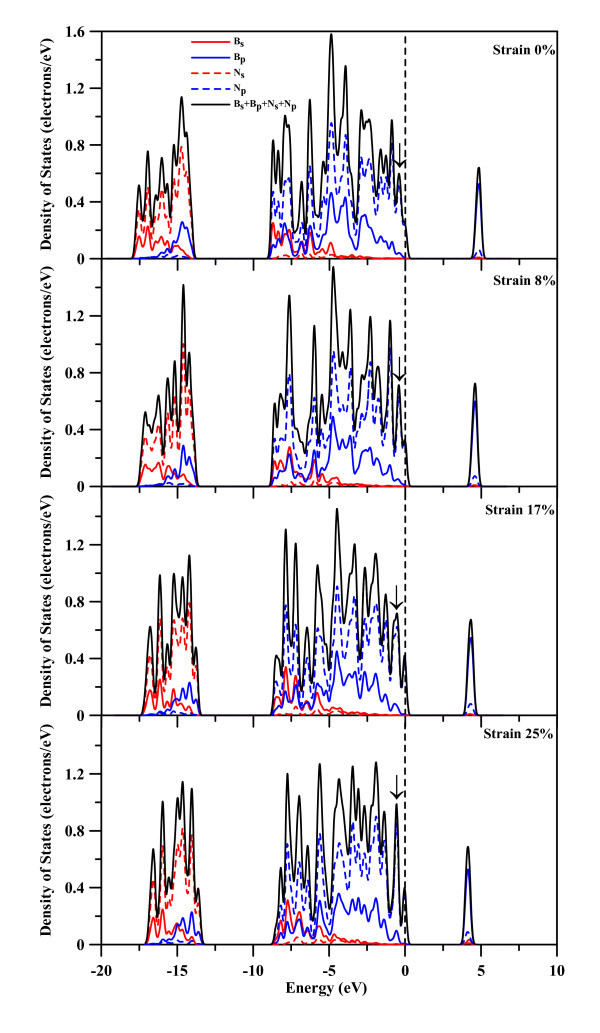
**PDOS profiles of B63 and N61 atoms**.

The partial density of states (PDOS) profiles for B68 and N35 atoms of the (8,0) BNNT and for B63 and N61 atoms of the (5,5) BNNT, as shown in Figures 7 and 8, respectively, are further studied to demonstrate the strain effect on the electronic structures of BNNTs. Figure 7a,b,c,d,e shows the PDOS of s and p orbitals of B68 and N35 atoms as well as the summation of these orbitals for the (8,0) BNNT. At strain of 0, there is no contribution to the total DOS from B68 2s and N35 2s orbitals around the Fermi level. It should be noted that the total DOS strength of empty states near Fermi level mainly comes from N35 2p electron and to a lesser degree B68 2p electron. The N35 2p orbital contributes more to the total DOS of occupied states near the Fermi level, and grabs electron from nearby B atoms. Moreover, the LUMO mainly comes from the B68 2p orbital and to a lesser degree N35's 2p orbital. Consequently, N atoms have negative charges and B atoms possess positive charges, which can be seen in Figure 6. At strain of 5%, the unoccupied state is split into two states, resulting in a significant decrease in the HOMO-LUMO gap when the strain is larger than 5%, as shown in Figure 2a. When the strain increases from 5 to 13%, the relative strengths of two split states become more dramatic, which can be seen in Figure 7b,c,d. At strain of 21%, both the occupied and unoccupied states display a significant left-shift and the two split unoccupied states merge into one unoccupied state, as shown in Figure 7e. The contribution from the B68 2p to the HOMO becomes less significant when the strain becomes larger, which can be seen at the peak indicated by arrows in Figure 7a,b,c,d,e. This reveals that N atoms will grab more electrons from B atoms when the strain becomes larger, and B and N atoms become more ionic, as was shown in Figure 6.

Figure 8a,b,c,d shows the PDOS of s and p orbitals of B63 and N61 atoms as well as the summation of those orbitals for the (5,5) BNNT. At strain of 0, there is almost no contribution to the total DOS from 2s orbitals of B63 and N61 around the Fermi level. The N61 2p orbital contributes more to the DOS of occupied states near the Fermi level, and grabs electron from nearby B atoms. Consequently, N atoms have negative charges and B atoms possess positive charges, as was shown in Figure 6. For the empty states, the total DOS strength mainly comes from the B63 2p electrons and to a lesser degree the N61 2p electron. As the strain increases to 8, 17, and 25%, the occupied states undergo a slight right-shift toward the Fermi level and the unoccupied states left-shift, resulting in a decrease of the HOMO-LUMO gap, which can be seen from Figure 2b. During the tensional process, the unoccupied state is not split into two states.

**Figure 9:**
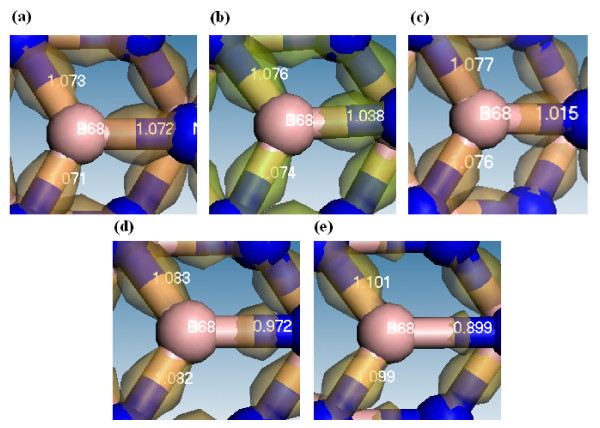
**Deformation density and Mayer bond orders are shown for boron on (8,0) BNNT at different strains**. The iso-value is 0.15. **(a) **Strain = 0%, **(b) **strain = 5%, **(c) **strain = 8%, **(d) **strain = 13%, **(e) **strain = 21%.

**Figure 10:**
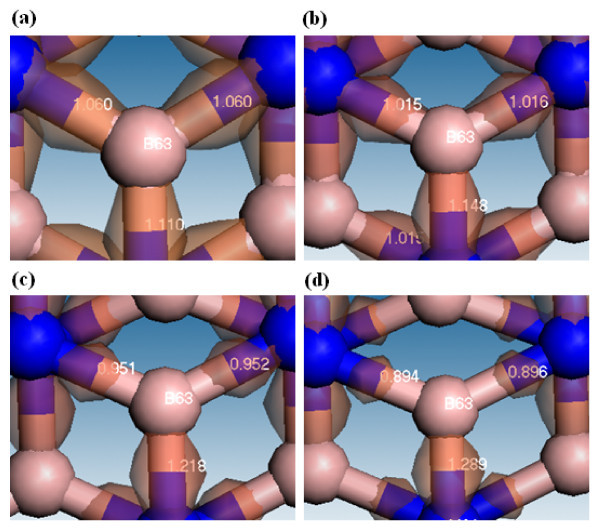
**Deformation density and Mayer bond orders are shown for boron on (5,5) BNNT at different strains**. The iso-value is 0.15. **(a) **Strain = 0%, **(b) **strain = 8%, **(c) **strain = 17%, **(d) **strain = 25%.

The electron differences at the iso-value of 0.15 and the Mayer bond orders (BO) of three B-N bonds at different strains for (8,0) and (5,5) BNNTs are shown in Figures 9 and 10. The electron difference is defined as the electron density distribution of BNNT minus the electron density distributions of isolated B atoms and isolated N atoms which constitute this BNNT. The value of the Mayer bond order between two atoms is very close to the corresponding classical bond number between these two atoms, and the detailed introduction of Mayer bond order can be found in Mayer's study [39]. The BO values are calculated within the first nearest neighbor atoms around a referenced atom, and this value becomes very small when the distance between the reference atom and its nearest neighbor atom is beyond the stable bond length. In Figure 9a, the distribution of positive iso-value around the B68 atom indicates that the extra electron will be accumulated between the B-N bond after the B and N atoms form the (8,0) BNNT at strain of 0. The BO values of two slanted B-N bonds are very close to that of the B-N bond parallel to the axial direction, indicating that the bond strengths of these two bond types are very close. Although the summation of the three BO values decreases from 3.216 to 3.099 as the strain continuously increases from 0 to 21%, the BO value of slanted bonds gradually increases from about 1.073 to 1.101, indicating the bonding strength will slightly increase under the larger strain. However, the BO of the B-N bond parallel to the axial becomes smaller at larger strains. The increase and decrease in the BO values for the slanted and parallel bonds become more considerable as the strain becomes larger than 5%, which is consistent with the variation of HOMO-LUMO gaps shown in Figure 2a. As the strain increases from 0 to 21%, the distributions of electron differences along the slanted bonds become wider, whereas that of the parallel bond turns out to be narrower. According to the result of the Mulliken charge analysis shown in Figure 6, B and N atoms become more ionic under the larger strain. Although the electrons transfer more from B atoms to N atoms at larger strain, the electron accumulation along the slanted bonds will become more significant.

In Figure 10a, the distribution of positive iso-value around the B63 atom indicates that the extra electron will accumulate between the B-N bond after the B and N atoms form the (5,5) BNNT at strain of 0. The BO values of two slanted B-N bonds are slightly smaller than that of the B-N bond normal to the axial direction, indicating that the slanted bond strength is slightly weaker than that of the bond normal to the axial direction. The summation of three BO values decreases from 3.23 to 3.079 as the strain continuously increases from 0 to 25%, but the BO value of the normal bond gradually increases from 1.110 to 1.289, indicating the bonding strength of the normal bond will slightly increase under the larger strain. However, the BO values of two slanted bonds become smaller at larger strains. As the strain increases from 0 to 25%, the distributions of electron differences along the slanted bonds become narrow, whereas that of the normal bond turns out to be wider, indicating that the electron accumulation along the slanted bonds will become more significant when the BNNT is under larger strain.

## Conclusion

This study utilizes DFT calculation to address the influence of axial tensions on the electronic properties of (8,0) zigzag and (5,5) armchair BNNTs. Although the stress-strain profiles indicate the mechanical properties of these two BNNTs are very similar, the variations of electronic properties at different uni-axial strains are drastically different. At strain lower than 5%, the HOMO-LUMO gap of (8,0) BNNT remains at a constant value, but decreases at a larger strain. For the (5,5) BNNT, the gap monotonically decreases when the strain becomes larger. The changes in nanotube geometries, PDOS of B and N atoms, B and N charges also indicate the uni-axial deformation definitely influences the electronic properties of (8,0) and (5,5) BNNTs.

## Abbreviations

Au-BNNT: Au-decorated boron nitride nanotube; BNNT: boron nitride nanotubes; CNTs: carbon nanotubes; DFT: density functional theory; DSPP: density functional semi-core pseudo-potentials; GGA: generalized gradient approximation; PDOS: partial density of states; PW91: Perdew-Wang 1991; SWBNNTA: single-walled boron nitride nanotube arrays; SWNTs: single-walled carbon nanotubes.

## Competing interests

The authors declare that they have no competing interests.

## Authors' contributions

TWL carried out the density functional theory simulation and performed the data analyze. YCW drafted the manuscript and participated in its design. SPJ participated in the design of the study and conceived of the study. All authors read and approved the final manuscript.
